# Associations Between Subjective Age, Contact With Older Adults, and Age Stereotypes Among Younger Adults

**DOI:** 10.1093/geroni/igaa057.1037

**Published:** 2020-12-16

**Authors:** Maria Kurth, Robert Intrieri

**Affiliations:** 1 Oregon State University, Corvallis, Oregon, United States; 2 Western Illinois University, MACOMB, Illinois, United States

## Abstract

Subjective aging (SA) has generally focused on middle-age and older adults in relation to physical and psychological health for the last 70 years (see Barak & Stern, 1986). Kornadt et al. (2019) recently called for more research examining: co-development of age stereotypes and SA, and this association across the lifespan. Literature examining SA and age stereotypes among younger adults is limited and suggests that age stereotypes are not directly associated with SA (Packer & Chasteen, 2006). Increased contact with older adults, however, is associated with less ageist attitudes (Bousfield & Hutchinson, 2010). This study examined SA and the associations between contact frequency and ageism. The sample consisted of 467 undergraduate students (Mage = 21.48, SDage = 2.63). Subjective age was assessed by asking How old do you feel compared with others your age?, and was scored on a 5-point scale from younger all the time (5) to older all the time (1). Ageism was assessed with the Aging Semantic Differential (ASD), which contains four factors. Results showed significant effects across felt age for contact frequency (F(4, 406) = 3.841, p = .004). Results for the ASD factors were mixed with Autonomy and Integrity showing significant effects for SA (F(4, 405) = 2.763, p = .027; F(4, 405) = 2.773, p = .027 respectively). Instrumentality and Acceptance were nonsignificant. Results suggested feeling older all the time is related to more contact, but more negative attitudes- this increased contact might providing priming for more ageist attitudes (Eibach et al., 2010).

